# Choroidal vascularity index in health and systemic diseases: a systematic review

**DOI:** 10.1186/s40942-024-00607-8

**Published:** 2024-11-18

**Authors:** Mehrdad Motamed Shariati, Sahel Khazaei, Mariye Yaghoobi

**Affiliations:** Eye Research Center, Khatam Al-Anbia Eye Hospital, Mashhad University of Medical Sciences, Gharani Boulevard, Mashhad, Iran

**Keywords:** Choroidal vascularity index, EDI-OCT, Inflammatory, Metabolic, Physiological, Systemic disease, Vascular layer

## Abstract

**Background:**

The choroid, a highly vascular structure within the eye, is significantly influenced by various systemic conditions. The advent of enhanced depth optical coherence tomography has improved our ability to evaluate choroidal pathophysiology. The choroidal vascularity index (CVI), a noninvasive and reliable tool, serves as an effective means of assessing the choroidal vascular structure. Recent studies have increasingly focused on exploring CVI alterations under different systemic conditions. This study aims to provide a comprehensive summary of the latest research findings in this area.

**Methods:**

A systematic literature review was conducted on October 1, 2023, using two databases, MEDLINE (via PubMed) and Scopus. Search terms were tailored specifically for each database to ensure a thorough exploration of relevant literature. The studies identified were qualitatively assessed, with particular emphasis on outcomes related to CVI and choroidal thickness.

**Results:**

A total of 48 studies were included in the review, encompassing a diverse range of systemic conditions such as diabetes, central nervous system disorders, cardiovascular diseases, autoimmune disorders, and infectious diseases. Notable reductions in CVI were observed in diabetic retinopathy, autoimmune diseases, and neurodegenerative disorders. Additionally, the review highlighted variations in CVI values related to the severity of systemic diseases, indicating its potential use as a biomarker for disease progression.

**Conclusion:**

This review highlights the significant correlation between variations in the choroidal vascularity index and diverse systemic conditions affecting hemodynamics. An enhanced understanding of CVI provides deeper insights into the pathophysiological mechanisms underlying these disorders and positions CVI as a promising biomarker for early detection and monitoring. Nevertheless, its clinical utility warrants careful assessment. Future research should address the potential limitations of CVI to fully capitalize on its diagnostic and prognostic potential.

**Supplementary Information:**

The online version contains supplementary material available at 10.1186/s40942-024-00607-8.

## Background

The choroid is a vascular layer located between the retinal pigment epithelium and lamina fusca of the sclera [1]. Choroid vessels are surrounded by stromal tissue, which comprises melanocytes, nerves, connective tissues, and extracellular fluid [2]. The vascular layer is composed of three layers, from internal to external, with an increased luminal diameter. The choriocapillaris is the innermost layer, the Sattler's layer has medium vessels in the middle, and the Haller's layer has large vessels in the outer layer [2]. The choroid has the highest blood flow per unit of body weight. 95% of the blood flow in the eye passes through the uvea, while the choroid receives more than 70% [3].

The choroid plays an important role in ocular health owing to its vascularity, and any disease affecting the vasculature could affect the choroidal vasculature [4].

Quantitative assessment of the choroidal vasculature has always been a challenge with conventional imaging modalities, such as ultrasonography and indocyanine green angiography [4].

The retina can be scanned with cross-sectional, high-resolution images using optical coherence tomography, which is a non-contact, noninvasive imaging technique. Chorioretinal diseases are now more easily diagnosed and managed using this valuable imaging [5]. The choroid can be characterized in vivo and noninvasively using enhanced depth imaging, swept-source OCT [3], and OCT angiography [6]. Advancements in OCT imaging have improved our ability to examine the choroid and evaluate the choroidal vasculature in different conditions of health and disease [3]. While OCTA has emerged as a pivotal technological instrument for assessing the choroid and offers innovative and significant insights into the pathogenesis of various choroidal and retinal disorders, it faces certain limitations. Improvements in software and hardware have only partially mitigated the substantial constraints stemming from motion, segmentation errors, signal attenuation, and projection artifacts [6].

Previous studies have primarily focused on the total thickness of the choroid. Choroidal thickness is the distance between the outside of the RPE and the inside of the sclera and is defined as the subfoveal choroidal thickness [3]. Using this method, only the total choroidal vasculature is shown, and there is no separation between the stromal and luminal vascular components [7].

Recently, some studies have evaluated the choroid in more detail, including measuring the choroidal vascular diameter and analyzing choroidal layers [8]. For the first time, in 2013, Branchini et al. calculated the ratio of light/dark areas corresponding to luminal and stromal areas of the choroid (luminal choroidal area and stromal choroidal area) in healthy eyes. This method can be used to quantify the choroidal vessel density [9].

Agrawal et al. suggested the choroidal vascularity index (CVI), a new quantitative measure calculated as the ratio of LCA to total choroidal area, for assessing choroidal vasculature in healthy and diseased eyes [7]. Previous studies have revealed that choroidal thickness is affected by temporary hemodynamic changes. Moreover, CT does not specify which part of the choroid, luminal area, or stromal tissue is affected by systemic diseases. It seems that CVI is a more reliable index of choroidal vasculature.

This paper aims to systematically review the literature regarding the changes in CVI in health and systemic conditions affecting hemodynamics. Additionally, it examines the existing limitations of CVI and potential improvements that could facilitate broader utilization of this diagnostic modality.

## Methods

### Selection process

We conducted a comprehensive literature search of two databases, MEDLINE (via PubMed) and Scopus, using the following keywords “systemic health”, “diabetes mellitus”, “carotid artery diseases”, “atherosclerosis”, “kidney diseases”, “rheumatoid arthritis”, “systemic vasculitis”, “systemic inflammatory disease”, “hypertension”, “pregnancy”, “autoimmune diseases”, “obstructive sleep apnea syndrome”, “smoking” which were then matched with the primary search term “choroidal vascularity index”, “CVI”. Reference lists of the selected reports were also screened manually. Grey literature was searched using the ProQuest Dissertations and Theses Global databases.

No time restrictions are imposed. The reviewers evaluated the studies according to the Preferred Reporting Items for Systematic Reviews and Meta-Analyses (PRISMA) 2020 Guidelines [10].

**Fig. 1:**
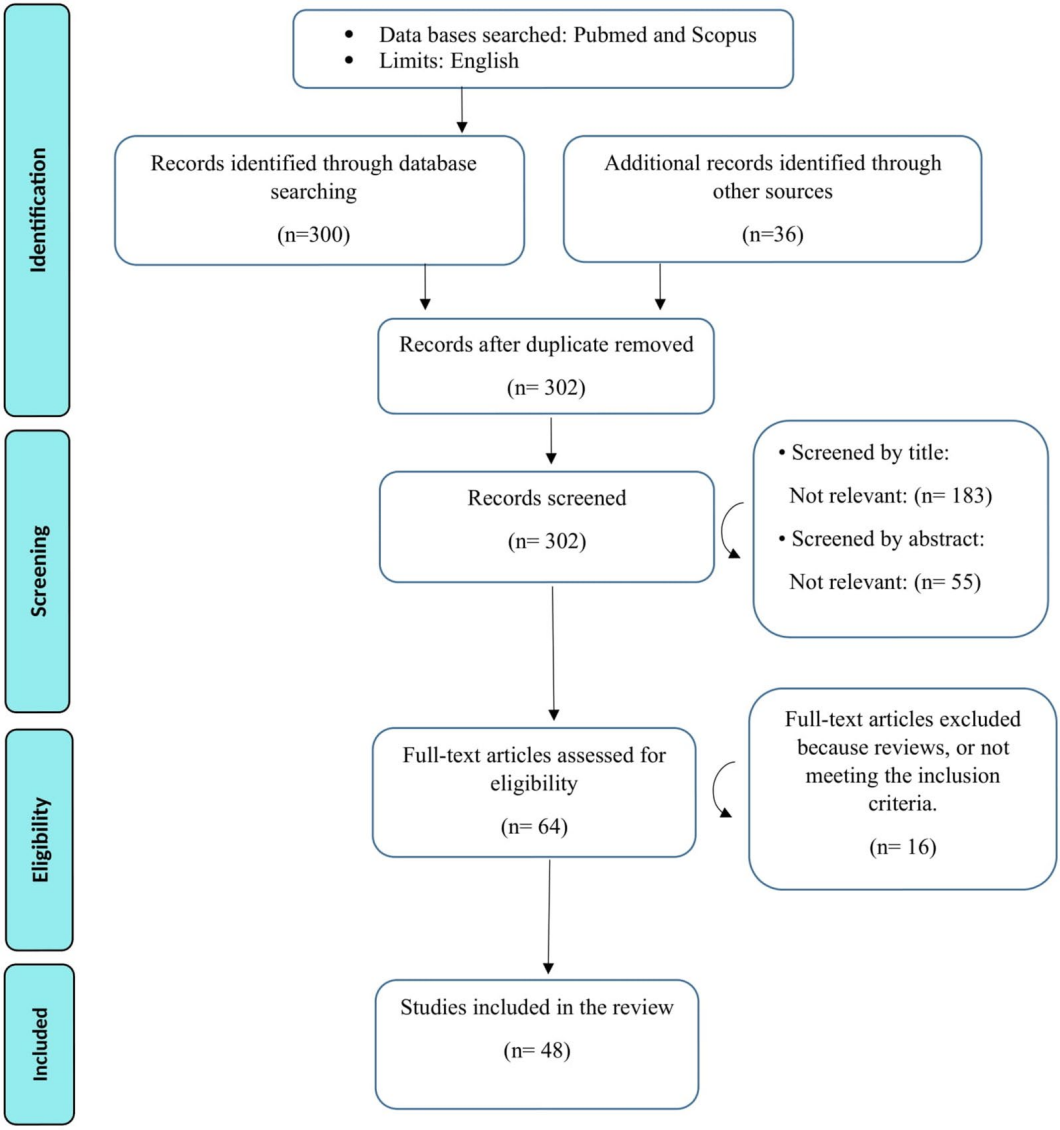
The study selection process

The inclusion criteria were: (1) Written in English, (2) evaluation of human subjects, (3) original research articles reporting outcomes of CVI calculation in macular areas in systemic conditions, and (4) clearly describing the database used and the number of images in various data sets. Exclusion criteria were: (1) reviews, systematic reviews, meta-analyses, case reports, case series, editorials, letters, and comments to the editor; (2) abstracts-only papers and those with unavailable full text; and (3) non-availability of original data. All records identified by the initial search were title-/abstract-screened. The full text of the remaining records was reviewed. References of those selected were screened as well. Two reviewers conducted independent searches and selected the records to be included. Quality assessment of the included records will be conducted using the National Institutes of Health Quality Assessment Tool for Observational Cohort and Cross-Sectional Studies [11]. Any disagreements were resolved through consensus. The selection process is shown in detail in Fig. 1.

### Data extraction

We extracted the following data from each study selected and compiled them in the standardized table (Additional file 1): detailed characteristics of the study (authors, year of publication, study design, medical profile, number of participants, methods, sample sizes), demographics (age, sex), and results of comparison between the CVI in specific systemic conditions and healthy population. Any unspecified or missing raw data were denoted as a ‘– (dash)’. The abbreviation “N.A. (not available)” was used in the tables if the data was inaccessible in the original article.

### CVI: definition and calculation

Non-invasive choroidal thickness and vasculature evaluation is possible using EDI-OCT, OCT-A, and SS-OCT imaging. The inner border of the choroid is the lower border of the hyperreflective band of the RPE/Bruch’s membrane layer and its outer border is the chorioscleral junction. As discussed earlier, the CVI is the proportion of the choroidal luminal area to the total choroidal area [12]. The amount of TCA depends on the desired segmentation diameter of the user. TCA and CVI values can differ depending on the retina's length, the imaging area (macula or peripapillary region), and the type of raster used (horizontal, vertical, or oblique). Usually, a region of 1.5 mm in length with foveal centrality in a horizontal raster scan is considered [7]. However, preceding investigations have evaluated diverse regions of interest, potentially hindering cross-study comparison of results.

Different methods have been proposed to calculate the CVI. The two main categories of calculation methods are manual calculations using image processing software and artificial intelligence-based methods. One of the most popular image-processing software packages for calculating CVI is ImageJ [13]. Automated measurement procedures have been introduced and applied in real-world clinical settings to address the limitations of manual techniques, which are time-consuming and prone to subjective error [14]. Nevertheless, these automated approaches also possess specific limitations that merit consideration in future investigations. For example, many are restricted to the macular area, and the utilization of diverse devices may impact the comparability of the outcomes.

## Results and discussion

### CVI in healthy subjects

Establishing normative values for CVI in healthy people has been the goal of several studies. In a study with a sample size of 345 healthy individuals with an average age of 61 years, Agarwal et al. evaluated CVI in the subfoveal region with a width of 1500 µm. The results of this study indicated a value of 65.61% ± 2.33% in healthy people [2]. Interestingly, CVI had a smaller coefficient of variation than subfoveal choroidal thickness. Furthermore, CVI value was only impacted by subfoveal CT, as opposed to subfoveal CT, which is influenced by some physiological parameters, including axial length, intraocular pressure, and age [2, 15, 16]. On the other hand, the findings of previous studies are inconclusive regarding the effect of axial length on CVI. While Goud et al. [17] reported no significant association between refractive error or axial length and CVI in normal individuals, other studies have found significantly lower CVI in highly myopic eyes, accompanied by choroidal thinning [18]. Proposed hypotheses for choroidal thinning in high myopia involve vascular and mechanical factors, including narrowing and loss of choroidal macro vessels, occlusion and ischemia of choroidal capillaries, and stretching of the sclera and choroidal stroma [19, 20]. Previous studies showed various results regarding how CVI is influenced by age [2, 16, 19, 21]. Some studies have found that the choroidal vascularity index does not change significantly with age in healthy individuals. In contrast, Ruiz-Medrano et al. reported that CVI is notably higher in people under 18 years of age [15]. Additionally, several studies have observed a significant association between CVI and age in the macular region, with a decline in luminal area and CVI as people advance in age [21–23]. This finding is consistent with prior histological evidence suggesting that the volume of choroidal cells and interstitial components decreases with aging [24]. In a study by Wang et al., no correlation between CVI and sex was identified in a cohort of healthy Chinese adults [22].

### Central nervous system disorders

Recently, the evaluation of the neurovascular structures of the retina has been proposed as a model for the assessment of neurological and psychiatric degenerative diseases. Regarding the common embryonic origin, similar blood supply system, neuronal network structure, and similar neurotransmitters, the retina has been proposed as a model for investigating neuropsychiatric diseases [25]. In addition, transparent ocular media enable accurate investigation of retinal neurovasculature using optical imaging methods such as OCT [26]. However, most previous studies have investigated the changes in macular thickness profile and retinal capillary densities with OCT angiography in CNS disorders in both domains of neurologic and psychiatric diseases [27]. With the improvement of OCT imaging methods and the use of ImageJ image processing software, attention has recently been paid to the evaluation of choroidal circulation changes in neurological and psychiatric diseases. Various mechanisms have been proposed to explain the possible use of choroidal circulation assessments in diseases involving the CNS. The common pathways of embryonic brain and eye development can lead to mirror changes in the choroid in neurodevelopmental disorders. In addition, previous studies have shown that an imbalance in neurotransmitters, such as glutamate and dopamine, leads to changes in choroidal circulation [28]. Another possible influencing mechanism is inflammatory processes. Neuroinflammation plays an important role in many diseases involving the CNS, such as MS, Parkinson’s disease, and psychosis, which could lead to changes in the vascular pattern of the choroid [29, 30]. Furthermore, neurodegeneration alters the homeostatic frame of CNS microcirculation, which can be investigated by choroidal imaging [31, 32].

#### Idiopathic normal pressure hydrocephaly

The results of a study that evaluated changes in choroidal vascular pattern in INPH indicated a significant increase in SFCT (260 ± 63 μm vs. 194 ± 40 μm, *p* < 0.001), TCA (2.55 ± 0.55 mm^2^ vs. 2.08 ± 0.46 mm^2^, *p* = 0.011), LCA (1.66 ± 0.30 vs. 1.38 ± 0.31 mm^2^, *p* = 0.020), and SCA (0.88 ± 0.20 vs. 0.70 ± 0.16 mm^2^) in patients compared to age-matched healthy subjects [33]. The choroidal vasculature empties into the vortex veins, and ultimately into the cavernous sinus. Decreased venous outflow from the choroid leads to choroidal engorgement, which might subsequently lead to increased choroidal thickness, as observed on OCT scans [33]. The increased stromal area in the choroid has been associated with stromal inflammation, as described previously [34, 35]. Given the hypothesized role of neurotoxin and cytokine accumulation in the cerebrospinal fluid, disruption of the blood–brain barrier, and microvascular dysregulation in the pathogenesis of iNPH, it is hypothesized that the observed increase in SCA could represent a subclinical OCT finding reflective of inflammation within the choroid, secondary to the accumulation of cytokines in the choroidal vasculature [36]. Additional research is needed to validate and deepen our comprehension of these findings, exploring the choroidal microstructure in the context of other neuroinflammatory disorders. The CVI did not differ significantly between individuals with iNPH and healthy controls, suggesting the ratio of total choroidal area to vascular area was maintained. However, the study reported no significant changes in these parameters 3 months after shunt surgery (281 ± 51.53 μm vs. 287 ± 42.80 μm, *p* = 0.223), likely due to the short follow-up period [33]. Further studies are needed to evaluate the role of CVI measurements in therapeutic response monitoring.

#### Parkinson’s disease

There is no consensus regarding the role of vascular factors in neurodegenerative processes. Some studies have attributed white matter changes observed in the MRI of Parkinson's patients to vascular disorders [37, 38]. Immunohistochemical evaluations have shown α-syn-GFP deposition around the retinal arterial vessels in a Parkinson's mouse model [39]. Furthermore, decreased superficial capillary density and destruction of the ganglion cell layer have been observed in the retina of Parkinson's patients [40, 41]. However, there is a lack of evidence regarding changes in choroidal vascular architecture in these patients. While some studies have shown choroidal thickening in the peripapillary area, another study has reported choroidal volume loss. Zhang et al. reported a significantly lower CVI in the early stages of Parkinson’s disease compared to healthy [42]. A decrease in CVI can have various causes, including α-syn-GFP deposits in the vessel wall and choroid stroma, vasomotor changes due to changes in the sympathetic and parasympathetic systems, and changes in the regional blood flow control mechanisms. These assumptions should be carefully evaluated in future studies.

#### Alzheimer’s disease

Retinal vascular network changes have been shown to decrease capillary density in AD [43, 44]. Studies on choroidal thickness change have not shown uniform results. Most previous studies have reported decreased choroidal thickness in patients with AD, attributed to a secondary vasoregressive process [45, 46]. Amyloid-β deposition and neurofibrillary tangle development result in neurotoxicity, loss of neurons and synapses, and vascular angiopathy 40 [47]. In a 2021 study, Robbins et al. observed decreased SFCT in AD patients but found that this measure did not significantly differ between AD and control eyes after adjusting for age, sex, and logMAR visual acuity. A limitation of this study was that the SFCT was not adjusted for axial length, which may have affected the results. They also found that the TCA and CLA were significantly higher among AD patients [48]. These results were consistent with the findings of a histopathological study by Asanad et al., which observed that choroidal thickness could vary regionally in AD and that increased choroidal vessel counts were associated with choroidal thickening [49]. Subclinical ischemia may initially result in increased choroidal vascular density during the early stages of the disease; however, the choroidal vascular parameters may diminish as the disease progresses to AD. Additionally, Robbin et al. reported a significantly lower CVI in patients with mild cognitive impairment than in healthy individuals [48].

#### Multiple sclerosis

Diseases such as MS that result in systemic inflammation and circulatory dysregulation can affect the choroidal vascular architecture. A recent study found no significant difference in choroidal thickness between eyes with MS-associated optic neuritis and healthy controls. However, the CVI was significantly lower in the MS group compared to healthy subjects (59.6 ± 3.72% vs. 61.7 ± 3.16%, *p* = 0.007). Additionally, the CVI demonstrated a statistically significant reduction in the optic nerve-affected eyes of multiple sclerosis patients relative to their unaffected contralateral eyes and the control group [50]. The observed choroidal changes cannot be solely attributed to the presence of MS and optic neuritis attacks, despite the possibility that the systemic treatments used to manage MS may have influenced these changes. However, the notable differences between the affected and unaffected eyes of MS patients provide evidence that the choroidal region is disproportionately impacted in the presence of optic neuritis history. These results strongly support the hypothesis that optic neuritis attacks have a significant effect on the vascular organization within the choroidal region. It is well-recognized that the pathophysiology of MS involves vascular disorganization processes. Sensitive perfusion-weighted MRI methods have recently shown that patients with MS exhibit reduced brain perfusion [51]. According to previous reports, there is an increase in the plasma levels of endothelin-1, a powerful vasoconstrictor peptide that might result in cerebral hypoperfusion [52]. The same mechanism could be suggested to justify the lower CVI in patients with MS. The findings indicate that quantitative evaluation of the choroid, particularly through CVI measurement, could provide valuable insights into the pathophysiological mechanisms underlying optic nerve pathology in MS.

### Autoimmune rheumatic diseases

Autoimmune and rheumatic diseases are a heterogeneous group of systemic inflammatory disorders with various manifestations. This group of disorders can affect the choroidal blood flow in different ways. Increased levels of inflammatory cytokines affecting the vessel wall, infiltration of inflammatory cells in the vessel wall, endothelial compensatory mechanisms to deal with inflammatory ischemia, vasomotor effects of anti-inflammatory drugs, toxic effects of immunomodulatory drugs, and direct involvement of ocular tissues are among these potential factors [4, 53]. Evaluation of the choroidal vasculature could potentially help determine systemic disease activity, response to treatment, adverse effects of drugs, and prognostication.

#### Behcet’s disease

BD is a systemic occlusive vasculitis involving multiple organs. Vascular tissue is a key target in BD, regardless of the presence of ocular inflammation, and many cases of vascular involvement are not clinically visible [54]. Previous studies have linked contradictory results to CT in individuals with nonocular BD. While most studies showed a significantly thicker choroid in non-ocular BD, the results of some studies depicted insignificant differences and even a significantly thinner choroid [55–57]. Simsek et al. investigated the choroidal vasculature in non-ocular BD in 2022. They showed that despite no significant difference in choroidal thickness between non-ocular BD and healthy subjects, the CVI was significantly lower in the BD group [58]. The theories put out on this subject include infiltration of stromal tissue (increased SA) and destruction of blood vessels as a result of occult vasculitis. In a recent study, researchers found that the CVI value was significantly lower in patients with ocular-involving BD than in healthy subjects. However, the difference in the CVI was not significant between non-ocular BD and healthy individuals [59]. These findings suggest that chronic uveitis affects choroidal microcirculation but not systemic inflammation.

#### Juvenile systemic lupus erythematosus

Systemic lupus erythematosus is a recurrent inflammatory disorder that affects multiple body systems. Several hypotheses have been proposed to explain the mechanism underlying chorioretinal involvement in SLE, including immune complex deposition, inflammation, and vaso-occlusive processes. A recent investigation found the choroid to be significantly thicker in SLE patients compared to healthy age- and sex-matched controls. Furthermore, the CVI was significantly lower in SLE patients with drusen-like deposits compared to healthy individuals 0.56 (0.54–0.59) versus 0.58 (0.57–0.59) (*p* = 0.018) [60]. This can be attributed to the fact that CVI is a relative measure calculated by dividing the luminal area by the total choroidal area. As an inflammatory disease, SLE primarily impacts the capillary systems and connective tissue [61]. As inflammation progresses, with the activation of inflammatory cytokines, antibodies, and cell infiltration, the stromal area expands through accumulation, while vascular occlusion due to the deposition of immune complexes and complement factors leads to a decrease in the diameter of the vascular lumen within the choroid [62]. Consequently, the CVI can decrease without a concurrent decline in absolute choroidal volume. Another study investigating CVI in juvenile SLE patients found no significant difference between the diseased and healthy groups, which was attributed to the concurrent inflammatory deposition in the stroma and vasodilation [61]. Additionally, patients with juvenile SLE may not exhibit substantial degenerative changes in the vasculature yet.

#### Juvenile idiopathic arthritis

Juvenile idiopathic arthritis is the most common chronic inflammatory disease associated with uveitis in pediatric patients. Uveitis is mostly anterior. However, the pathophysiology and prognosis of both ocular and systemic disorders may be better understood in light of alterations in the choroid and retina. Agin et al. in a study showed that the choroid was significantly thicker in JIA-associated uveitis and JIA with no uveitis compared to healthy subjects. The study found that in both JIA-associated uveitis and JIA without uveitis, the TCA, LA, and SA were increased compared to healthy controls [63]. The authors hypothesized that this indicates the presence of subclinical choroidal inflammation with the involvement of the stromal tissue in JIA patients. Furthermore, the CVI was significantly lower in JIA-associated uveitis compared to controls, suggesting more stromal involvement in JIA patients with uveitis. Importantly, the absence of significant differences in choroidal parameters between the acute and remission phases of JIA-associated uveitis implies that these alterations are related to the chronic, rather than acute, nature of the condition [63]. The increases in LA and SA may be attributed to the activation of the autoinflammatory cascade, driven by autoinflammatory molecules predominantly found in the choroidal interstitial tissue, which play a crucial role in the pathogenesis of the disease [64].

#### Polyarteritis nodosa

PAN is a systemic necrotizing vasculitis that involves small- and medium-sized vessels. In a recent study, researchers showed that the subfoveal choroid was significantly thicker in patients with PAN than in a healthy group (342 µm vs. 242 µm, *p* < 0.0001). In addition, choroidal SA and choroidal LA were significantly higher in the PAN group. However, CVI values were not significantly different [65]. The higher LA in PAN could be attributed to microaneurysms and increased vascular permeability due to vasculitis, while systemic inflammation causes an enlarged SA [66, 67]. The combination of these factors likely contributed to the observed increase in both LA and SA, while the choroidal vascular index remained stable within the studied groups. Changes in both LA and SA were linked to increased choroidal thickness [65].

### Diabetes mellitus

The choroid plays an important role in the development of DR. Prior research has demonstrated some changes in the choriocapillaries of diabetic eyes, including thickened basement membranes, capillary dropout, lumen narrowing, and arteriosclerotic alterations in the choroidal arteries. Identifying the choroid may aid in measuring the progression of DR, as diabetic choroidopathy may be an important component of DR development [68]. The fenestrated vascular structure of the choroid and its deep position under the RPE layer make assessment challenging [69]. Choroidal thickness is frequently used as an alternate indicator of choroidal vascularity to associate it with the stage of DR or to predict the progression of retinopathy in diabetic patients. However, previous investigations have produced inconsistent results [70–73]. These conflicting findings indicate that choroidal thickness is poorly correlated with the development of DR or DME. This poor association may be attributed to the fact that choroidal thickness is affected by several systemic and ocular factors, such as age, axial length, intraocular pressure, diurnal variations, and blood pressure [74]. CVI is regarded as a reliable method of assessing choroidal vascular structure because it is less affected by ocular or systemic factors [75].

Previous research indicated that people with DM and DR had significantly lower CVI levels than the general population [69, 75–78]. It was shown by Obadă et al. that SFCT, TCA, LA, and SA were similar among normal subjects, subjects with DM without DR, and subjects with DM and NPDR without DME. CVIs were significantly lower in NPDR patients compared to DM patients without DR [76]. In a similar study, Tan et al. demonstrated that patients with DM had decreased CVI without changes in choroidal thickness [69]. Eyes with mild-to-moderate NPDR also had a lower CVI than healthy control eyes [78]. The study by Kim et al. clarified that in the early stages of DR, SFCT and CVI diverged. Comparing the mild and moderate NPDR group with the no DR group, SFCT was greater and CVI was significantly lower. This suggests that stromal thickening is responsible for choroid thickening in early stages of DR, and not vascular components. Even without DR, diabetic patients' eyes had a significantly lower CVI value than healthy controls [75]. In a study of patients with diabetes without DR, it was found that their macular CVI of Haller's layer, but not Sattler's layer, was lower compared with healthy control eyes, even though CTs, RTs, or volumes of the two groups were not significantly different. In addition, patients with diabetes with disease duration longer than 5 years had lower choroidal volumes and subfoveal CVIs of Sattler's layer but not CTs or Haller's layer CVIs, compared to patients who had diabetes for less than 5 years [79]. Larger choroidal veins appear to be the primary target of DC in the initial stages of DR, preceding medium-sized arterioles [79]. The deficit of choroidal blood flow may be a precursor of DR, according to findings in an animal model [80]. As diabetes progresses, the diameter and density of the choroidal vessels decrease, and choroidal blood flow decreases before retinopathy appears [81]. When the disease progresses to severe NPDR or PDR, the SFCT and CVI start to decrease. Choroidal hypoxia and choriocapillary loss may cause choroidal thinning as DR progresses. It's unknown if choroidal alterations are the cause or consequence of retinopathy. Considering that CVI is significantly lower in diabetics even without DR, ischemic changes in the choroidal vasculature may be the initial incident in diabetes before the development of DR. It has been noted in previous reports that CVI decreases as DR severity increases [75, 82, 83]. Hence, CVI may be helpful for early detection and monitoring of the progress of DR.

DME, the leading cause of severe vision loss in diabetics, affects both outer and inner retinas and can occur at any stage of DR [84]. According to Gupta et al., CVI was significantly lower in DME with DR eyes than in controls with healthy eyes [82]. Changes in the choroid in eyes with DR and DME are dynamic, and they may be key to the pathogenesis of retinal changes in these conditions. CVI analysis may provide insight into the pathogenesis of DME.

Marques et al. reported that the CVI of patients with DR requiring treatment (intravitreal injections and/or photocoagulation) was lower than that of normal subjects or DR patients without treatment, regardless of changes to LA, SA, or CT [85]. However, in another report, the mean CVI of the PRP-treated group did not show a significant difference from either the PDR group or the severe NPDR group [75]. CVI changes may be more affected by the severity of DR than by the treatment itself.

The measurement of CVI in diabetic patients could also be used to predict diabetic vascular complications other than DR. A desire exists to develop reliable non-invasive indicators for diabetic nephropathy so that invasive methods such as renal biopsy can be limited. Due to the similar physiological and pathological characteristics of the renal and retinal circulations, the presence of DR frequently indicates the occurrence of DN [86]. Patients with DN stage III (based on pathologic findings) had a significantly lower CVI than those with DN stages IIa or IIb, according to Han et al. [83]. Therefore, CVI seems to decrease with increasing severity of kidney disease as well as increased severity of microvascular lesions in the eyes. In addition, this study showed that CVI was superior to DR for the prediction of DN in terms of sensitivity and specificity. The CVI (measured from subfoveal choroid within a width of 1500 μm) cutoff was 63.13%. Accordingly, CVI may serve as a more appropriate routine screening marker for diabetic patients with renal damage than DR [83]. Diabetes patients with CKD are at increased risk of cardiovascular morbidity and mortality due to hyperphosphatemia [87]. A higher level of serum phosphorus, even in those without CKD, is related to a higher risk of myocardial infarction [88]. A negative correlation was reported between the CVI of naive eyes with DR and serum phosphorus levels. It is possible that reduced CVI in eyes with DR is associated with vascular calcification of the choroidal vessels or the cardiovascular system [78].

A study by Kim et al. examined the relationship between systemic arterial stiffness by measuring cardio-ankle vascular index and microvascular alterations in the choroid and retina in DM. They found that the CAVI and the CVI were negatively correlated in DR, suggesting that arteriosclerosis is associated with choroidal vascular changes [89].

There is only one study comparing CVI and other related quantitative choroidal parameters in pediatric patients with DM type 1 and healthy controls. The importance of diagnosing DR early is heightened by the fact that type 1 DM begins at a younger age than type 2 DM and leads to retinopathy earlier than type 2 DM. In the type 1 DM group, TCA, LA, and SA values were significantly higher. Type 1 DM patients had lower LA/SA and CVI values, but not significantly. CVI and LA/SA exhibited a negative correlation with DM duration. To verify these results, long-term investigations with large patient groups are required [90].

### Thyroid associated ophthalmopathy

Currently, there is evidence that thickening of the choroid occurs during the active phase of inflammatory and autoimmune diseases such as thyroid-related ophthalmopathy [53]. Due to venous congestion in the choroid of TAO patients, it is difficult to demonstrate structural alterations histopathologically. The choroid consists of blood vessels enclosed by an extracellular matrix. CT changes did not reveal which components were affected more and in what proportions. As well, an increase in one component may be counterbalanced by a decrease in the other component. Because of these reasons, CT may not accurately represent choroidal structural changes [91]. To understand the mechanism behind choroidal thickening in TAO, a closer analysis of choroidal stromal and vascular structures may be helpful. In terms of evaluating choroidal parameters in TAO, there is limited research with contradictory results [91, 92].

According to Loiudice et al., despite similar SFCT, the CVI was higher in eyes with TAO compared to healthy controls [92]. There have been previous reports of increased CT in eyes with TAO, however [93–95]. The heterogeneity of the population studied may contribute to inconsistent results. TAO patients and healthy subjects showed no difference in CVIs in another study with a larger sample size [91]. It was claimed that the modifications in stromal and vascular structures are proportionally equal in TAO; therefore, CVI may not be significantly affected. It is more likely that chronic inflammation or an alteration in the stromal component due to circulating antibodies, glycosaminoglycan deposition, or fibroblast activation is responsible for this characteristic stromal involvement rather than only mechanical compression causing vascular congestion. Such pathophysiologic processes may reflect TAO's inflammatory nature [91]. Identifying how these choroidal parameters change longitudinally during the progression of TAO, as well as assessing the potential effects of medical or surgical treatments on the choroid, requires further research with large and homogeneous samples.

### Nonalcoholic fatty liver disease

Recent studies have demonstrated that NAFLD affects many other systems other than the liver [96]. Cardiovascular diseases are more prevalent in patients with NAFLD, and CVD is the leading cause of death among these patients [97]. A number of subclinical atherosclerosis markers have been correlated to NAFLD, including increased carotid intima-media thickness, calcification of coronary arteries, impaired vasodilation, and arterial stiffness [98]. According to Avcı et al., patients with NAFLD had a statistically significant lower CVI than those without ultrasonographic fatty livers [99]. This result implies that NAFLD is a multisystem disease that changes the microvascular system.

### Obesity and weight loss

In obese individuals, nitric oxide levels are reduced, resulting in impaired vessel dilation. A higher BMI is also associated with higher levels of certain vasoconstrictor molecules, such as endothelin-1 and angiotensin II [100]. The decreased blood flow in the choroid may be caused by low NO levels and high vasoconstrictors in obese patients. According to a study by Agarwal et al., patients with obesity have lower retinal and choroidal vascular parameters, such as CVI and capillary density index, compared to normal participants. However, neither the mean retinal nor choroidal thickness of these participants differed significantly from normal controls. It is interesting to note that after bariatric surgery, choroidal thickness values and arteriovenous ratio increased. This may be due to the altered hemodynamics. However, there was no change in parameters such as CVI and CDI despite bariatric surgery [101]. A modest sample size, a relatively short follow-up period, as well as numerous confounding factors, limit the generalizability of the findings of this study.

### Obstructive sleep apnea syndrome

Multiple studies have analyzed the effects of Obstructive sleep apnea syndrome on CT, but their results are inconsistent [102]. Altinel et al. calculated the choroidal structural areas and found that the OSAS group had greater mean values for LA, SA, and TCA than the control group prior to continuous positive airway pressure therapy. However, these differences were not statistically significant. Endothelial dysfunction and oxidative stress-induced inflammation in OSAS can account for these findings. Due to the higher percentage of SA involvement in the OSAS group, CVI was found to be significantly lower prior to CPAP therapy. Following 12 months of consistent use of CPAP therapy, there was a significant decrease in SA, which can be attributed to a decrease in stromal edema caused by the therapy. Hence, CPAP treatment increased CVI, suggesting that CVI could be a valuable metric for monitoring OSAS patients [103]. Prospective research with greater sample sizes is necessary on this topic.

### Carotid cavernous fistula

CCF is frequently misdiagnosed or delayed in diagnosis due to the disease's various symptoms and scarcity. Early detection and treatment of CCF is critical for minimizing morbidity. While a carotid angiogram is the gold standard for diagnosing CCF, clinicians may benefit from noninvasive imaging to corroborate their initial suspicion before scheduling more expensive and invasive investigations. EDI-OCT is a non-invasive and available tool that may assist in the diagnosis and follow-up of CCF. A study comparing choroidal and retinal thickness, CVI, and tortuosity index between patients with anterior CCF, posterior CCF, and healthy controls demonstrated that patients with A-CCF had significantly higher CVI and therefore higher luminal and luminal-to-stromal ratio values than the control group. The A-CCF group showed decreased stromal area percentage values. Conversely, the P-CCF had parameters similar to the control group [104]. It is speculated that high venous pressure from CCF leads to increased luminal elements within the choroid, resulting in increased choroidal thickness. Similarities in choroidal measures between P-CCF and the control group may be due to P-CCF's drainage to the inferior petrosal sinus rather than the ophthalmic vein. Thus, these fistulas do not result in choroidal congestion [104]. Arat et al. found a significant reduction in choroidal thickness, CVI, and TI measures after successfully closing the fistula. They proposed that CVI and TI, along with choroidal thickness measures, can serve as a noninvasive first-line follow-up approach for assessing the closure of the fistula via endovascular treatment or spontaneous resolution. In addition, evaluation of choroidal measures, including CVI, revealed subclinical involvement of the clinically unaffected eye in patients with unilateral CCFs with anterior drainage [105].

### Carotid artery stenosis

Considering the retina as an extension of the brain, OCT has been suggested as a non-invasive, high-resolution technique for assessing the pathophysiology of CAD [106]. Currently, the effect of carotid stenosis on choroidal circulation is poorly understood. Comparing CAS patients and healthy controls, Kwapong et al. found that CVI and choroidal vascular volume were reduced. According to this study, changes in the retina and choroid may be able to identify hemodynamic changes in CAS patients and predict stroke risk. There was not a significant difference in CVI between the ipsilateral and contralateral eyes of CAS patients, however [107].

### Coronary artery disease

There is a disparity between the findings from studies comparing SFCT in patients with various stages of CAD [108, 109]. A study investigating CVI in CAD patients and those without CAD found that CVI was lower in the triple vessel disease group compared to other stages of the disease. However, no significant difference existed between the no CAD group and the 1–2 vessel disease group. Additionally, male sex, hypertension, and high CVI were the only variables among various traditional risk factors identified to be significantly associated with triple-vessel disease [109]. This suggests that CVI may be helpful for detecting severe CAD but not for identifying early changes in the coronary arteries. However, the limited sample size and presence of confounding factors in this study call for further research to confirm the hypothesis.

### Systemic hypertension

Systemic hypertension, a prevalent chronic condition characterized by elevated blood pressure, exerts a significant influence on the cardiovascular system, including the microvasculature of the eye. The choroid, with its rich vascular network, is particularly susceptible to the deleterious effects of sustained high blood pressure. The relationship between systemic hypertension and choroidal structure is multifaceted and influenced by various factors. Some research indicates that hypertension may contribute to a decrease in CT, potentially due to the compression of choroidal vessels from elevated intravascular pressure or hypertensive microangiopathy and arteriolar sclerosis [110, 111]. A recent study has demonstrated that treatment-naïve hypertensive adults exhibit significantly reduced TCA, LA, and CVI compared to healthy individuals [112]. This may be attributed to the vasoconstrictor effects of the sympathetic nervous system in response to elevated systemic blood pressure, which can result in a thinner choroid and diminished choroidal hemodynamic parameters. Further research is warranted to elucidate the complex interplay between hypertension, CVI, and potential confounding factors. Additionally, investigating the impact of antihypertensive medications on CVI could provide valuable insights into their protective effects on ocular microcirculation.

### Renal hemodynamics

The choroidal vasculature changes are associated with renal hemodynamics. The CVI had a significant association with the estimated glomerular filtration rate [109]. Due to alterations in the body's fluid content, hemodialysis may modify the thickness and volume of the choroids. Shin et al. measured CVI before and after hemodialysis and found that the LA, SA, TCA, and SFCT decreased significantly after hemodialysis, but there was no significant difference in CVI. Furthermore, these studies revealed no significant differences between the DM and non-DM groups. Systemic fluid retention before hemodialysis may be accompanied by the accumulation of fluid in the intravascular and interstitial components of the choroid. The decrease in choroidal thickness, LA, and SA following hemodialysis could be attributed to the elimination of intravascular and interstitial fluids due to the increased transcapillary colloid osmotic gradient. Thus, the CVI may remain unchanged following hemodialysis due to reductions in both LA and SA. These findings show that CVI may be a more stable indicator of vascular health than choroidal thickness [113].

### Heart failure

Heart failure with a reduced ejection fraction is accompanied by peripheral vasoconstriction, which keeps the vital tissues properly perfused and oxygenated [114]. Compensatory vasoconstriction may affect the choroidal arteries, resulting in reduced SFCT98. A recent study examined 52 eyes of 26 patients with HFrEF (whose left ventricular ejection fraction was less than 40%) and 64 eyes of 32 healthy individuals. The study found that the choroidal vascular index (CVI) was decreased in the HFrEF group, indicating that both the stroma and lumen of the choroid were shrinking, which may be linked to a reduction in choroidal thickness in HFrEF [115]. The choroid can regulate its blood flow in response to variations in ocular perfusion pressure. This ability appears to be modest but significant [116]. To find out if CVI can help patients with HFrEF in clinical practice, more long-term observations of a larger number of patients are required.

### COVID-19

Earlier studies have shown that COVID-19 can lead to a reversible decrease in CVI, indicating the disease's multiple reversible effects on ocular perfusion [117, 118]. During active COVID-19 infection, there is a significant decrease in choroidal vascularity and an increase in SA to vascular area ratio, likely due to choroidal stromal edema, endothelial inflammation, and vessel wall thickening. Micro-thromboembolic issues may also play a role [117]. Patients with moderate COVID-19 have lower mean CVI, but the values return to baseline levels within 4 months of remission. Monitoring of recovered patients reveals a dramatic CVI increase 1 month after symptom onset, followed by a return to baseline levels 3 months later. The stromal area shows the most noticeable changes, while the luminal area exhibits the greatest variation [118, 119]. However, a recent study found no significant CVI difference between COVID-19 patients and healthy subjects [120]. Further research is needed to fully understand the duration of COVID-19's impact on ocular vascular changes.

### CVI limitations and potential future advancements

The current body of literature surrounding the CVI reveals several important limitations that warrant further examination. A significant portion of existing studies has predominantly focused on Asian populations, often utilizing small sample sizes and limited follow-up durations. Consequently, the generalizability of CVI findings to a broader, more diverse global population remains uncertain. As familiarity with this metric increases, future studies are anticipated to adopt more robust methodologies, incorporating extended observation periods and larger, more heterogeneous participant groups that represent a diverse array of ethnic and geographic backgrounds. Another critical aspect affecting the reliability of CVI measurements is the quality of OCT imaging. Potential segmentation errors can introduce biases in CVI calculations, particularly when the choroidal–scleral interface is not clearly identifiable. Challenges such as shadows cast by retinal blood vessels can obscure choroidal structures during OCT imaging, resulting in inaccurate CVI readings. Although techniques like shadow compensation visualization have been proposed to improve the visibility of the choroid, further validation is needed to establish their efficacy in routine clinical practice [121]. Factors such as high myopia, ocular opacities, involuntary eye movements, and underlying pathological conditions can also significantly impact image quality and the subsequent accuracy of choroidal visualization [122].

Additionally, variations in the defined regions of interest across studies can impede cross-study comparisons of CVI results. The inconsistency in selection criteria for the areas assessed introduces further complications, limiting the ability to draw definitive conclusions about the CVI’s implications in different contexts. Addressing these biases and methodological inconsistencies will be crucial for advancing our understanding of CVI's role in ocular health and disease.

## Conclusion

This systematic review elucidates the correlation between changes in CVI and various systemic diseases affecting hemodynamics. The study of CVI can provide crucial insights into systemic disorders' pathophysiology. Moreover, CVI can be considered a valuable tool for early diagnosis and monitoring of several diseases. This insight may pave the way for improved clinical strategies in managing systemic diseases. However, it is important to acknowledge the limitations of CVI measurements. Future research should address the following: standardization of measurement techniques, the influence of physiological factors, and conducting longitudinal studies to understand the long-term effects of systemic diseases on choroidal vascularity and the potential implications for disease progression and treatment outcomes.

## Supplementary Information

**Additional file 1.**

## Data Availability

No datasets were generated or analysed during the current study.

## Abbreviations

A-CCF: Anterior carotid cavernous fistula
AD: Alzheimer’s disease
BD: Behcet’s disease
BMI: Body mass index
CAD: Coronary artery disease
CAS: Carotid artery stenosis
CAVI: Cardio-ankle vascular index
CCF: Carotid cavernous fistula
CKD: Chronic kidney disease
CNS: Central nervous system
CPAP: Continuous positive airway pressure
CT: Choroidal thickness
CVD: Cardiovascular diseases
CVI: Choroidal vascularity index
DC: Diabetic choroidopathy
DM: Diabetes mellitus
DME: Diabetic macular edema
DN: Diabetic nephropathy
DR: Diabetic retinopathy
EDI: Enhanced depth imaging
HFrEF: Heart failure with a reduced ejection fraction
INPH: Idiopathic normal pressure hydrocephaly
JIA: Juvenile idiopathic arthritis
LA: Luminal area
LCA: Luminal choroidal area
MRI: Magnetic resonance imaging
MS: Multiple sclerosis
NAFLD: Nonalcoholic fatty liver disease
NO: Nitric oxide
NPDR: Non-proliferative diabetic retinopathy
OCT: Optical coherence tomography
OSAS: Obstructive sleep apnea syndrome
PAN: Polyarteritis nodosa
P-CCF: Posterior carotid cavernous fistula
PDR: Proliferative diabetic retinopathy
PRP: Pan-retinal photocoagulation
ROI: Regions of interest
RPE: Retinal pigment epithelium
RT: Retinal thickness
SA: Stromal area
