# Supplementary material for: Choroidal vascularity index in health and systemic diseases: a systematic review
Source: Int J Retina Vitreous. 2024 Nov 18;10:87. doi: 10.1186/s40942-024-00607-8 (PMC11575059; doi:10.1186/s40942-024-00607-8)
Supplement: Supplementary file 1 — Additional file 1. [file 40942_2024_607_MOESM1_ESM.docx]

| **Table 1. Key papers included in this review** | | | | | | | | | | | |
| --- | --- | --- | --- | --- | --- | --- | --- | --- | --- | --- | --- |
| **S/N** | | **Author(year)** | **Study design** | **Setting** | | **Medical profile** | **Sample size** | | **Age (mean year)** | **Male gender (%)** | **CVI changes associated with the systemic disease** |
| 1 | | Kim (2019)(89) | Cross-sectional, hospital/clinic-based | South Korea | | DM type 2 with/ without systemic arterial stiffness (CAVI≥ 9) | No DR | 18 | 55.94 | 38.9 | ↓ CVI in the group with abnormal CAVI (≥ 9) Negative correlation of CAVI with CVI (r =−0.247) |
|  |  |  |  |  |  |  | NPDR | 71 | 60.42 | 47.9 |  |
|  |  |  |  |  |  |  | PDR | 24 | 56.96 | 20.8 |  |
| 2 | | Damian (2021)(123) | Prospective, hospital/clinic based | Romania | | DM type 1 or 2 vs Healthy | Healthy controls | 36 | 53.28 | 55.55 | ↑ average CVI in the NDR group ↓ CVI in the DR group versus control The correlation of RPE with CVI (r= 0.479) |
|  |  |  |  |  |  |  | No DR | 44 | 57.08 | 55.55 |  |
|  |  |  |  |  |  |  | DR | 27 | 57.83 | 55 |  |
| 3 | | Gupta (2018)(82) | Cross-sectional, hospital/clinic-based | India | | Treatment naïve DME vs Healthy | Healthy controls | 86 | 54.98 | 53.5 | ↓CVI in DME with DR eyes as compared to controls (63.89±1.89 vs 67.51±2.86) ↓CVI with worsening DR |
|  |  |  |  |  |  |  | DR with DME | 82 | 55.77 | 52.44 |  |
| 4 | | Obadă (2022)(76) | Cross-sectional, hospital/clinic-based | Romania | | DM vs Healthy | Healthy controls | 21 | 71.14 | 42.86 | ↓CVI in NPDR group compared to DM without DR |
|  |  |  |  |  |  |  | DM without DR | 20 | 71.75 | 40 |  |
|  |  |  |  |  |  |  | DM with NPDR without DME | 22 | 63.41 | 40.91 |  |
| 5 | | Sidorczuk (2022)(77) | Cross-sectional, hospital/clinic-based | Poland | | DR vs Healthy | Healthy controls | 76 | 55.73 | 46.2 | ↓CVI in the presence of DR (with or without DME) |
|  |  |  |  |  |  |  | DR with DME | 76 | 61.5 | 49 |  |
|  |  |  |  |  |  |  | DR without DME | 134 | 60.03 | 47.8 |  |
| 6 | | Marques (2022)(85) | Cross-sectional, hospital/clinic-based | Portugal | | DM vs non-diabetic | Non-DM | 73 | 72.2 | 41.8 | Significant ↓ CVI in Treated DR group compared to controls and non- treated DR Correlation between BCVA and CVI (r=− 0.362) Lower CVI in patients treated with PRP compared to other treatments |
|  |  |  |  |  |  |  | Non-DR | 39 | 71.3 |  |  |
|  |  |  |  |  |  |  | Non- treated DR | 52 | 66.7 |  |  |
|  |  |  |  |  |  |  | Treated DR | 154 | 66.7 |  |  |
| **Table 1. Continued** | | | | | | | | | | | |
| **S/N** | | **Author(year)** | **Study design** | **Setting** | | **Medical profile** | **Sample size** | | **Age (mean year)** | **Male gender (%)** | **CVI changes associated with the systemic disease** |
| 7 | | Foo (2020)(79) | Prospective, poulation- based | Singapore | | DM vs Healthy | Healthy controls | 74 | 50.8 | 55.4 | Significant ↓ of the macular CVI of Haller’s but not Sattler’s layer in DM without DR compared to healthy controls ↓ CVI of Sattler’s layers in eyes with >5 years of diabetes |
|  |  |  |  |  |  |  | DM without DR | 75 | 50.3 | 54.7 |  |
|  |  |  |  |  |  |  | DR | 18 | 53.6 | 55.6 |  |
| 8 | | Kim (2021)(78) | Cross-sectional, hospital/clinic-based | South Korea | | DM type 2 vs Healthy | Healthy controls | 30 | 59.1 | N. A | Significant ↓ CVI mild-to-moderate NPDR compared to healthy controls CVI of naïve eyes with DR negatively correlated with age (r= -0.312) and the serum levels of phosphorus (r= -0.422) and positively correlated with SFCT (r= 0.560) |
|  |  |  |  |  |  |  | No DR | 32 | 58.2 | 64.88 |  |
|  |  |  |  |  |  |  | Mild/Moderate NPDR | 33 | 58.7 |  |  |
|  |  |  |  |  |  |  | Severe NPDR | 34 | 55.9 |  |  |
|  |  |  |  |  |  |  | Treatment- naïve PDR | 32 | 49.6 |  |  |
| 9 | | Kim (2018)(75) | Cross-sectional, hospital/clinic-based | South Korea | | DM type 2 vs Healthy | Healthy controls | 45 | 57.47 | N. A | Significant ↓ CVI in diabetic eyes even without Dr compared to healthy controls Significant ↓ CVI in PDR group compared to healthy control, no DR, and mild/moderate NPDR groups |
|  |  |  |  |  |  |  | No DR | 30 | 57.5 | 43.24 |  |
|  |  |  |  |  |  |  | Mild/Moderate NPDR | 41 | 59.17 |  |  |
|  |  |  |  |  |  |  | Severe NPDR | 40 | 59.83 |  |  |
|  |  |  |  |  |  |  | PDR | 8 | 58.5 |  |  |
|  |  |  |  |  |  |  | PRP-treated DR | 35 | 54.09 |  |  |
|  |  |  |  |  |  |  | CSME | 31 | 59.1 |  |  |
| 10 | | Tan (2016)(69) | Prospective, hospital/clinic based | Singapore | | DM vs Healthy | Healthy controls | 38 | 70 | _ | Significant ↓ CVI in patients with DM as compared to controls |
|  |  |  |  |  |  |  | DM | 38 | 68.55 | _ |  |
|  | |  |  |  | |  |  |  |  |  |  |
| **Table 1. Continued** | | | | | | | | | | | |
| **S/N** | | **Author(year)** | **Study design** | | **Setting** | **Medical profile** | **Sample size** | | **Age (mean year)** | **Male gender (%)** | **CVI changes associated with the systemic disease** |
| 11 | | Duran (2023)(90) | Prospective, hospital/clinic based | | Turkey | DM type 1 children vs Healthy | Healthy | 46 | 12.89 | 58.69 | Negative correlation between duration of DM and LA/SA (r= -0.377) and CVI (r= -0.377) No significant difference between the groups in terms of the CVI |
|  |  |  |  |  |  |  | DM type 1 | 43 | 13.01 | 60.46 |  |
| 12 | | Han (2022)(83) | Cross-sectional, hospital/clinic-based | | China | DM with proteinuria | DN | 90 | 54.2 | 75.6 | ↓ CVI in the severe NPDR and PDR groups compared to no DR and mild/moderate NPDR groups Lower CVI in DN stage III compared with DN stages IIa and IIb Preferable sensitivity and specificity of the CVI as compared with DR for diagnosing DN ( 84% (71%–94%) and 95% (88%–99%), respectively) |
|  |  |  |  |  |  |  | Non-DN | 144 | 57.6 | 62.5 |  |
| 13 | | Khalilipur (2023)(115) | Cross-sectional, hospital/clinic-based | | Iran | HFrEF vs Healthy | Healthy controls | 64 | 51 | 62.5 | ↓ CVI in HFrEF compared to controls but not significantly |
|  |  |  |  |  |  |  | HFrEF group | 52 | 53 | 76.92 |  |
| 14 | | Seo (2022)(109) | Cross-sectional, hospital/clinic-based | | South Korea | CAD vs Healthy | No CAD | 42 | 61.6 | 57 | ↓CVI in the triple vessel disease group compared to those in the other groups Significant association of CVI with eGFR , SFCT, and the presence of the triple vessel disease |
|  |  |  |  |  |  |  | 1-2 vessel disease | 31 | 64.3 | 71 |  |
|  |  |  |  |  |  |  | Triple vessel disease | 17 | 66.5 | 88 |  |
|  |  |  |  |  |  |  |  |  |  |  |  |
| 15 | | Inam (2019)(104) | Cross-sectional, hospital/clinic-based | | Turkey | CCF vs Healthy | Healthy controls | 19 | _ | _ | ↑CVI in the A-CCF group compared to controls |
|  |  |  |  |  |  |  | CCF | 19 | 54.27 | 52.63 |  |
| **Table 1. Continued** | | | | | | | | | | | |
| **S/N** | | **Author(year)** | **Study design** | **Setting** | | **Medical profile** | **Sample size** | | **Age (mean year)** | **Male gender (%)** | **CVI changes associated with the systemic disease** |
| 16 | | Arat (2022)(105) | Prospective, hospital/clinic based | Turkey | | Unilateral A-CCF | Unilateral A-CCFs | 22 | 55.4 | 36.36 | Significant ↑ in CVI after successful closure of the fistula compared to first presentation ↓CVI in D-CCFs compared to the I-CCF group at presentation Similar CVIs in both groups At the last follow-up visit |
| 17 | | Kwapong (2022)(107) | Cross-sectional, hospital/clinic-based | China | | Carotid artery stenosis | Carotid artery stenosis (Ipsilateral and contralateral eyes) | 74 | 63.95 | 86.48 | Relative cerebral blood volume showed a significant correlation with CVI (r= 0.11) |
| 18 | | Valsecch (2023)(33) | Prospective, hospital/clinic based | Italy | | Idiopathic normal pressure hydrocephalus vs Healthy | Healthy controls | 18 | 76.26 | 55.6 | ↑ TCA, LCA, and SCA in iNPH patients before ventriculo‑peritoneal surgery compared to the control group No diferences in the CVI between iNPH patients and controls |
|  |  |  |  |  |  |  | iNPH | 18 | 77.16 | 72.2 |  |
| 19 | | Zhang (2022)(42) | Cross-sectional, hospital/clinic-based | China | | Parkinson’s disease vs Healthy | Healthy controls | 200 | 56.63 | 50 | Significant ↓ CVI in PD patients compared with healthy controls |
|  |  |  |  |  |  |  | Parkinson’s disease | 100 | 57.92 | 50 |  |
| **Table 1. Continued** | | | | | | | | | | | |
| **S/N** | | **Author(year)** | **Study design** | **Setting** | | **Medical profile** | **Sample size** | | **Age (mean year)** | **Male gender (%)** | **CVI changes associated with the systemic disease** |
| 20 | | Balci (2021)(50) | Prospective, hospital/clinic based | Turkey | | MS with history of unilateral ON vs Healthy | Healthy controls | 30 | 33.4 | 60 | ↓Mean CVI in the the eyes affected with optic neuritis attack in patients with multiple sclerosis than their healthy fellow eye ↓CVI of both the MSON and MSNON compared to the controls |
|  |  |  |  |  |  |  | Affected eyes (ON attack) of the MS patients | 20 | 33.25 | 65 |  |
|  |  |  |  |  |  |  | Unaffected eyes of MS patients | 20 |  |  |  |
| 21 | | Loiudice (2021)(92) | Cross-sectional, hospital/clinic-based | Italy | | TAO vs Healthy | Healthy controls | 40 | 37.45 | 26.25 | ↑CVI in patients with TAO No association between SFCT or CVI and TCA, clinical activity score, exophthalmometric value, Inami value, diplopia status |
|  |  |  |  |  |  |  | TAO | 40 | 39.3 |  |  |
| 22 | | Yeter (2021)(91) | Cross-sectional, hospital/clinic-based | Turkey | | TAO vs Healthy | Healthy controls | 53 | 46.9 | 43.39 | No statistically significant difference between the groups for CVI, CSI, CVI1500, CSI1500, and SA/LA A significant correlation of CVI1500 and CSI1500 with exophthalmometry (r= -0.21 and r= 0.21, respectively) and clinical activity score (r =-0.35 and r= 0.35) |
|  |  |  |  |  |  |  | TAO | 53 | 46.7 | 43.39 |  |
| 23 | | Xuan (2023)(19) | Cross-sectional, population- based | China | | Healthy | Healthy subjects | 556 | 56.4 | 55.04 | Older age and longer AL were significantly associated with a lower CVI |
| 24 | | Wang (2023)(22) | Cross-sectional, hospital/clinic-based | China | | Healthy | Healthy subjects | 1566 | 43.62 | 50 | A significant negative correlation of CVI with age Sex had no effect on CVI |
|  | **Table 1. Continued** | | | | | | | | | | |
|  | | | | | | | | | | | |
| **S/N** | | **Author(year)** | **Study design** | **Setting** | | **Medical profile** | **Sample size** | | **Age (mean year)** | **Male gender (%)** | **CVI changes associated with the systemic disease** |
| 25 | | Agrawal (2016)(2) | Cross-sectional, population- based | Singapore | | Healthy | Healthy subjects | 365 | 61.53 | 44.92 | CVI was less variable than SFCT (coeffcient of variation for CVI was 3.55 vs 40.30 for SFCT) Higher CVI was associated with thicker SFCT, but not associated with most physiological variables. |
| 26 | | Singh (2019)(124) | Prospective, hospital/clinic-based | India | | Healthy | Healthy subjects | 24 | 26 | 33.33 | The mean CVI showed a signifcant diurnal variation in the temporal quadrant of the peripapillary region  SF-CVI variations correlated well with SBP variations (r=0.61) |
| 27 | | Nivison‑Smith (2020)(21) | Cross-sectional, hospital/clinic-based | Australia | | Healthy | Healthy subjects | 106 | 48.24 | 51.88 | Decrease in CVI with age/ A decrease in CVI from the ages of 33–43 years at a rate of 0.7–2.7% per decade |
| 28 | | Wei (2019)(125) | Cross-sectional, hospital/clinic-based | India | | Cigarette smoking | Non- smokers | 44 | 36.56 | 100 | Lower CVI in smokers compared to non-smokers |
|  |  |  |  |  |  |  | Smokers | 39 | 41.79 | 100 |  |
| 29 | | Suzuki (2021)(126) | Prospective, hospital/clinic-based | Japan | | Pregnancy | Pregnant | 24 | 38 | 0 | No significant difference in the CVI throughout pregnancy or the postpartum period |
| 30 | | Azuma (2021)(127) | Prospective, hospital/clinic-based | Japan | | Pregnancy | Control | 26 | 35.5 | 0 | ↑ CVI in pregnancy as compared with control |
|  |  |  |  |  |  |  | Pregnant | 32 | 35.9 | 0 |  |
| **Table 1. Continued** | | | | | | | | | | | |
| **S/N** | | **Author(year)** | **Study design** | **Setting** | | **Medical profile** | **Sample size** | | **Age (mean year)** | **Male gender (%)** | **CVI changes associated with the systemic disease** |
| 31 | | Shim (2021)(128) | Cross-sectional, hospital/clinic-based | South Korea | | Pre-eclampsia | Pre-eclampsia | 61 | 34.3 | 0 | CVI were not related with urine protein–creatinine ratio |
| 32 | | Agarwal (2020)(101) | Prospective, hospital/clinic-based | India | | Obesity | Obese adults | 60 | 46.47 | 78.33 | ↑ mean CVI among normal subjects compared to participants with obesity |
| 33 | | Kim (2018)(75) | Cross-sectional,  hospital/clinic-based | South Korea | | Human Leukocyte Antigen-B27-Associated Uveitis | unilateral HLA-B27-associated uveitis patients | 45 | 38.89 | 60 | ↑ mean CVI and choroidal thickening in acute HLA-B27-associated uveitis that subsequently decreased after uveitis resolution |
| 34 | | Simsek (2022)(58) | Cross-sectional, hospital/clinic-based | Turkey | | Behcet disease | Healthy controls | 35 | 33.5 | 60 | Lower CVI values in the macula and temporal, nasal, and inferior sectors of the peripapillary area in the BD group compared with controls |
|  |  |  |  |  |  |  | Behcet disease | 32 | 34.6 | 62.5 |  |
| 35 | | Kukan (2022)(60) | Observational cohort | Switzerland | | Systemic Lupus Erythematosus | Healthy controls | 51 | 57.7 | 47.1 | Lower CVI values in patients with DLD compared to eyes of the healthy subjects No significant difference in CVI comparing eyes of patients with DLD to eyes of patients without DLD |
|  |  |  |  |  |  |  | SLE with DLD | 16 | 41.2 | 33.3 |  |
|  |  |  |  |  |  |  | SLE without DLD | 16 | 40.6 | 31.3 |  |
| 36 | | Ağın (2019)(61) | Cross-sectional, hospital/clinic-based | Turkey | | Juvenile systemic lupus erythematosus | jSLE | 21 | median age: 13 | N.A | CVI of jSLE patients were similar to those of the healthy control |
| 37 | | Ağın (2022)(63) | Cross-sectional, hospital/clinic-based | Turkey | | Juvenile rheumatoid arthritis | JIAU | 28 | 10 | 14.28 | Lower CVI in JIAU patients compared to JIAN patients and healthy controls |
|  |  |  |  |  |  |  | JIAN | 65 | 11.8 | 38.46 |  |
| **Table 1. Continued** | | | | | | | | | | | |
| **S/N** | | **Author(year)** | **Study design** | **Setting** | | **Medical profile** | **Sample size** | | **Age (mean year)** | **Male gender (%)** | **CVI changes associated with the systemic disease** |
| 38 | | Baytaroğlu (2020)(65) | Cross-sectional, hospital/clinic-based | Turkey | | Childhood Polyarteritis Nodosa and adenosine deaminase-2 deficiency | PAN | 11 | 8 | 45.45 | Similar CVI in both groups (PAN and DAD2) Similar CVI scores of PAN and DADA2 patients under treatment and from healthy controls |
|  |  |  |  |  |  |  | DAD2 | 4 | 6 | 75 |  |
| 39 | | Liu (2018)(35) | Cross-sectional,  hospital/clinic-based | China | | Vogt–Koyanagi–  Harada Disease | Healthy controls | 40 | 37.3 | 57.5 | ↑ CVI in VKH patients compared to healthy controls |
|  |  |  |  |  |  |  | VKH patients | 40 | 36.1 | 57.5 |  |
| 40 | | De Bernardo (2021)(129) | Observational case–control | Italy | | Celiac | Healthy controls | 67 | 40.67 | 44.77 | Celiac patients have a thicker choroid than healthy subjects, regardless of the AL Due to a proportional increase in both the vascular and stromal components, CVI was the same as controls |
|  |  |  |  |  |  |  | Celiac disease | 74 | 40.78 | 28.37 |  |
| 41 | | Avcı (2023)(99) | Prospective, hospital/clinic-based | Turkey | | Non-alcoholic fatty lier disease | Fatty liver (-) | 88 | 41 | 27.27 | ↓ CVI in NAFLD group comparing to those without fatty liver |
|  |  |  |  |  |  |  | Fatty liver (+) | 104 | 46 | 53.84 |  |
| 42 | | Altinel (2022)(103) | Cross-sectional, hospital/clinic-based | Turkey | | Obstructive sleep apnea | Healthy controls | 26 | 48.65 | 76.9 | ↓ CVI in OSAS patients before treatment with CPAP compared to healthy controls After 12 months of regular CPAP therapy, the mean CVI value increased significantly |
|  |  |  |  |  |  |  | OSAS | 22 | 45.5 | 68.2 |  |
| 43 | | D’Aloisio (2023)(120) | Cross-sectional, hospital/clinic-based | Italy | | COVID-19 infection | COVID-19 patients | 46 | 50.2 | N.A | CVI did not show significant differences between the cases and healthy controls |
| **Table 1. Continued** | | | | | | | | | | | |
| **S/N** | | **Author(year)** | **Study design** | **Setting** | | **Medical profile** | **Sample size** | | **Age (mean year)** | **Male gender (%)** | **CVI changes associated with the systemic disease** |
| 44 | | Bayram (2021)(117) | Prospective, hospital/clinic-based | Turkey | | COVID-19 infection | COVID-19 patients | 53 | 50.2 | 52.83 | ↓ CVI in the active phase of infection |
| 45 | | Abrishami (2022)(119) | Prospective, hospital/clinic-based | Iran | | COVID-19 infection | COVID-19 recovered patients | 34 | 34.6 | N.A | Significant ↑ CVI after one month and returned to baseline after three months of recovery from COVID-19 |
| 46 | | Kocamış Ö (2022)(118) | Cross-sectional, hospital/clinic-based | Turkey | | COVID-19 infection | Healthy controls | 34 | 37.2 | 41.2 | ↓ the mean TCA, SA, LA and CVI in COVID-19 patients ↑the choroidal structural parameters and CVI 4 months after remission as compared with the baseline measurements in the patients with COVID-19 |
|  |  |  |  |  |  |  | COVID-19 patients | 32 | 35.5 | 56.2 |  |
| 47 | | Shin (2019)(113) | Prospective, hospital/clinic-based | South Korea | | End-stage renal disease | Diabetes mellitus group | 18 | 58.2 | 50 | ↓ LA, SA, TCA, and SFCT after hemodialysis No significant change in the CVI after hemodialysis |
|  |  |  |  |  |  |  | Non-diabetes mellitus group | 14 | 54.1 | 28.6 |  |
| 48 | | Aşıkgarip (2022)(112) | Prospective, hospital/clinic-based | Turkey | | Treatment- naive hypertension | Systemic hypertension | 50 | 51.2 | 46 | ↓ mean TCA, LA, and CVI in the patient group in comparison to controls |
|  | |  |  |  | |  | Healthy control | 50 | 50.9 | 50 |  |

**Abbreviations**: **A- CCF**, carotid- cavernous fistula with anterior drainage; **ADHD**, attention- deficit/ hyperactivity disorder; **BCVA**, best-corrected visual acuity; **CAD**, coronary artery disease; **CAVI** ,cardio-ankle vascular index; **CCF**, carotid- cavernous fistula; **CPAP**, continuous positive airway pressure; **CSI**, choroidal stromal index; **CSI1500**, choroidal stromal index within 1500 mm of central fovea; **CSME**, clinically significant macular edema; **CVI** ,choroidal vascularity index; **CVI1500**, choroidal vascularity index within 1500 mm of central fovea; **D- CCF** ,direct carotid- cavernous fistula; **DAD2**, deficiency of adenosine deaminase-2; **DLD**, drusen-like deposits; **DM** ,diabetes mellitus; **DME**, diabetic macular edema; **DN**, diabetic nephropathy; **DR** ,diabetic retinopathy; **eGFR**, estimated glomerular filtration rate; **HFrEF**, heart failure with reduced ejection fraction; **I-CCF**, indirect CCF; **TCA** ,total choroidal area; **iNPH**, Idiopathic normal pressure hydrocephalus; **JIAN**, juvenile rheumatoid arthritis without associated uveitis; **JIAU**, juvenile rheumatoid arthritis with associated uveitis; **jSLE**, Juvenile systemic lupus erythematosus; **LA**, luminal area; **SA**, stromal area; **LCA** ,luminal choroidal area; **MPH**, Methylphenidate; **NPDR**, non- proliferative diabetic retinopathy; **OSAS**, obstructive sleep apnea; **PAN**, polyarthritis nodosa; **PD**, Parkinson’s disease; **MS**, multiple sclerosis; **PDR**, proliferative diabetic retinopathy; **PRP**, pan-retinal photocoagulation; **RPE**, retinal pigment epithelium; **SBP**, systolic blood pressure; **SCA** , stromal choroidal area; **SFCT**, subfoveal choroidal thickness; **SF-CVI**, subfoveal choroidal vascularity index; **SLE**, systemic lupus erythematous; **TAO**, thyroid- associated ophthalmopathy; **VKH**, Vogt–Koyanagi–Harada Disease
